# Supplementary material for: Episodic release of CO2 from the high-latitude North Atlantic Ocean during the last 135 kyr
Source: Nat Commun. 2017 Feb 22;8:14498. doi: 10.1038/ncomms14498 (PMC5322501; doi:10.1038/ncomms14498)
Supplement: Supplementary Information — Supplementary Figures, Supplementary Tables and Supplementary References [file ncomms14498-s1.pdf]

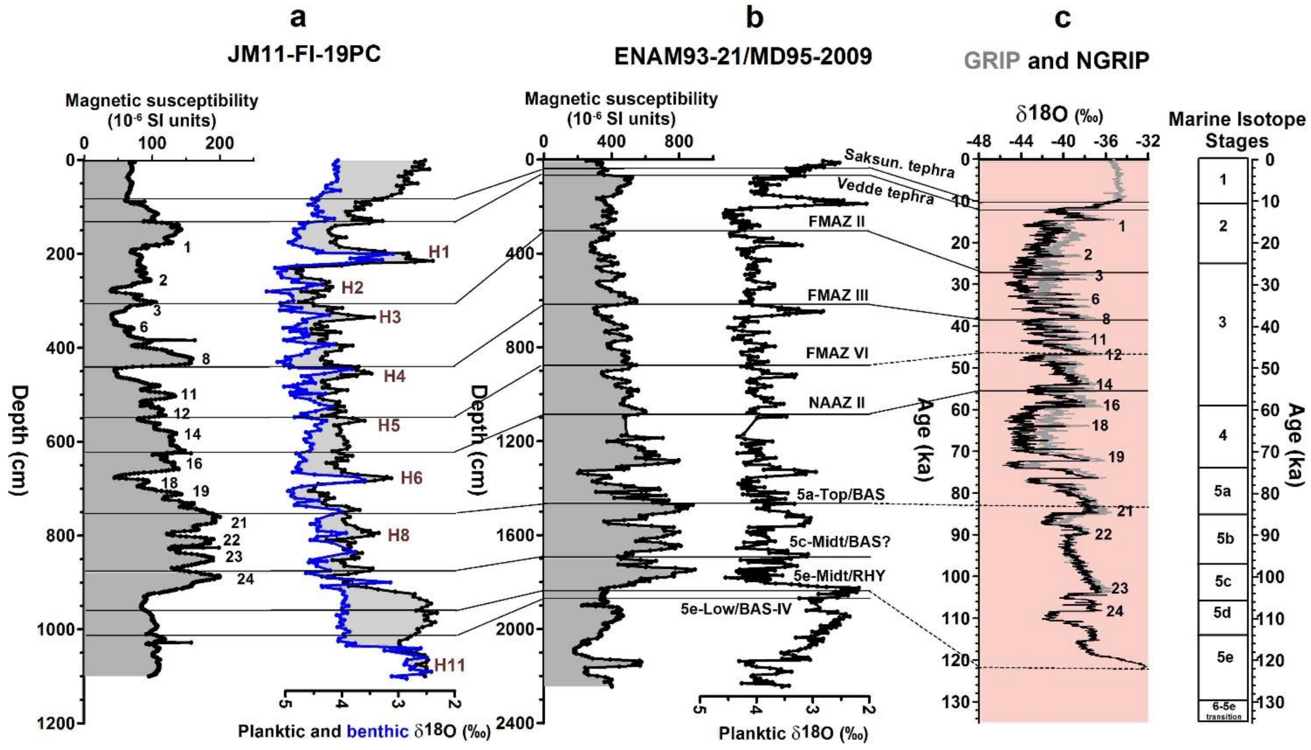

Supplementary Figure 1: Correlation of sediment core JM-FI-19PC to nearby marine sediment cores and Greenland ice cores. **(a)** Correlation of magnetic susceptibility as well as planktic and benthic $\delta^{18}O$ in JM-FI-19PC (refs. 1–3) to **(b)** nearby sediment cores ENAM93-21 and MD95-2009 (refs 4, 5) and **(c)** $\delta^{18}O$ values from Greenland ice cores[6,7]. Solid black horizontal lines mark tephra layers identified in both marine sediments and in ice cores[7]. Tephra layers not yet confirmed in the ice cores and their potential location in ice core records are shown by dashed black lines. Interstadial numbers (black labels), Heinrich events (brown labels) and Marine Isotope Stages (right hand panel) are indicated.

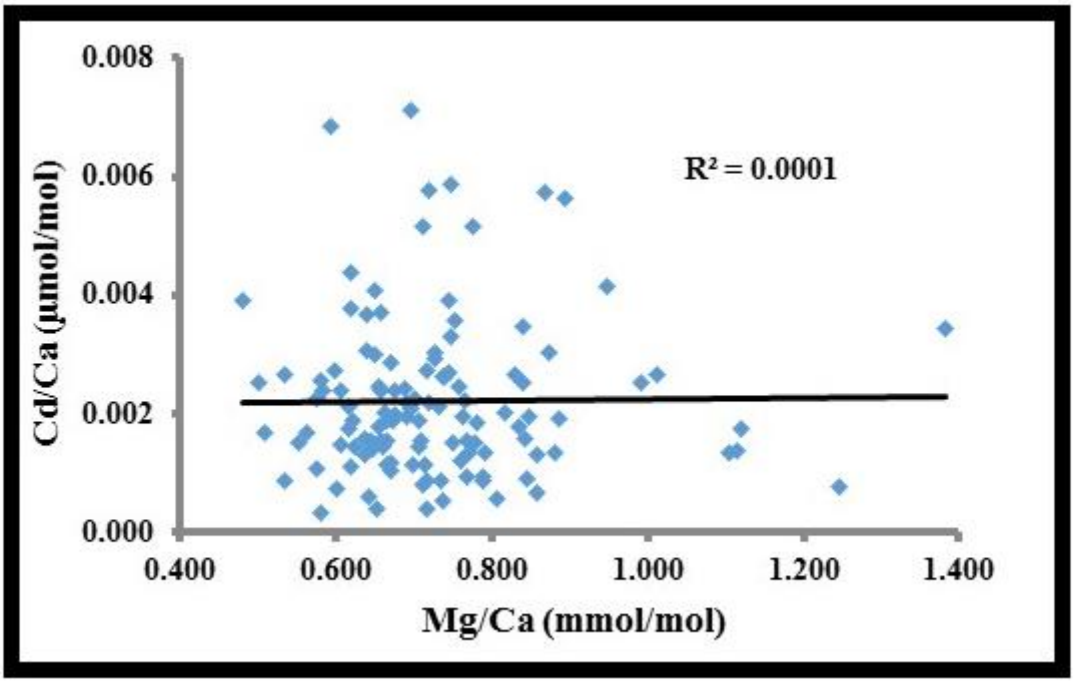

Supplementary Figure 2. Cross-plot of Cd/Ca and Mg/Ca. The absence of a correlation between both proxies indicates our downcore Cd/Ca data are not affected by temperature variations.

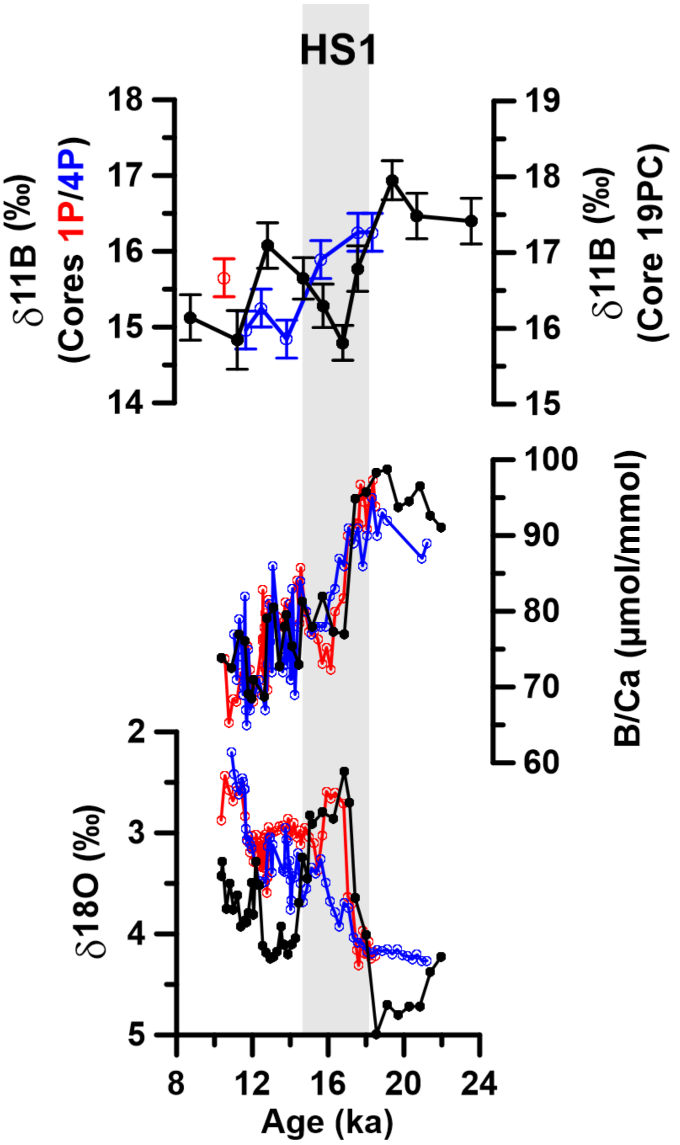

Supplementary Figure 3. δ^{11}B, B/Ca and δ^{18}O measured in *N. pachyderma* from sediment core JM-FI-19PC (1179 m water depth; black) from the southern Norwegian Sea (this study; refs 1, 2) and sediment cores RAPiD-10-1P (1237 m water depth; red) and RAPiD-15-4P (2133 m water depth; blue) from south of Iceland[8,9]. We slightly modified the age models in Yu *et al.*[9] for Heinrich Stadial (HS) 1 by aligning the records from south of Iceland to JM-FI-19PC using the start of the decrease in δ^{18}O in *N. pachyderma* as a tuning marker. Note that we used a thermal ionization mass spectrometer to measure δ^{11}B, whereas Yu *et al.*[9] used a multicollector inductively coupled plasma mass spectrometer, which yields ~1‰ lower δ^{11}B values for the same samples. Inter-instrumental offsets are well known for δ^{11}B analyses, but consistent relative variations are obtained between different laboratories and techniques[10,11]. Thus, we used a separate y-axis for each δ^{11}B dataset.

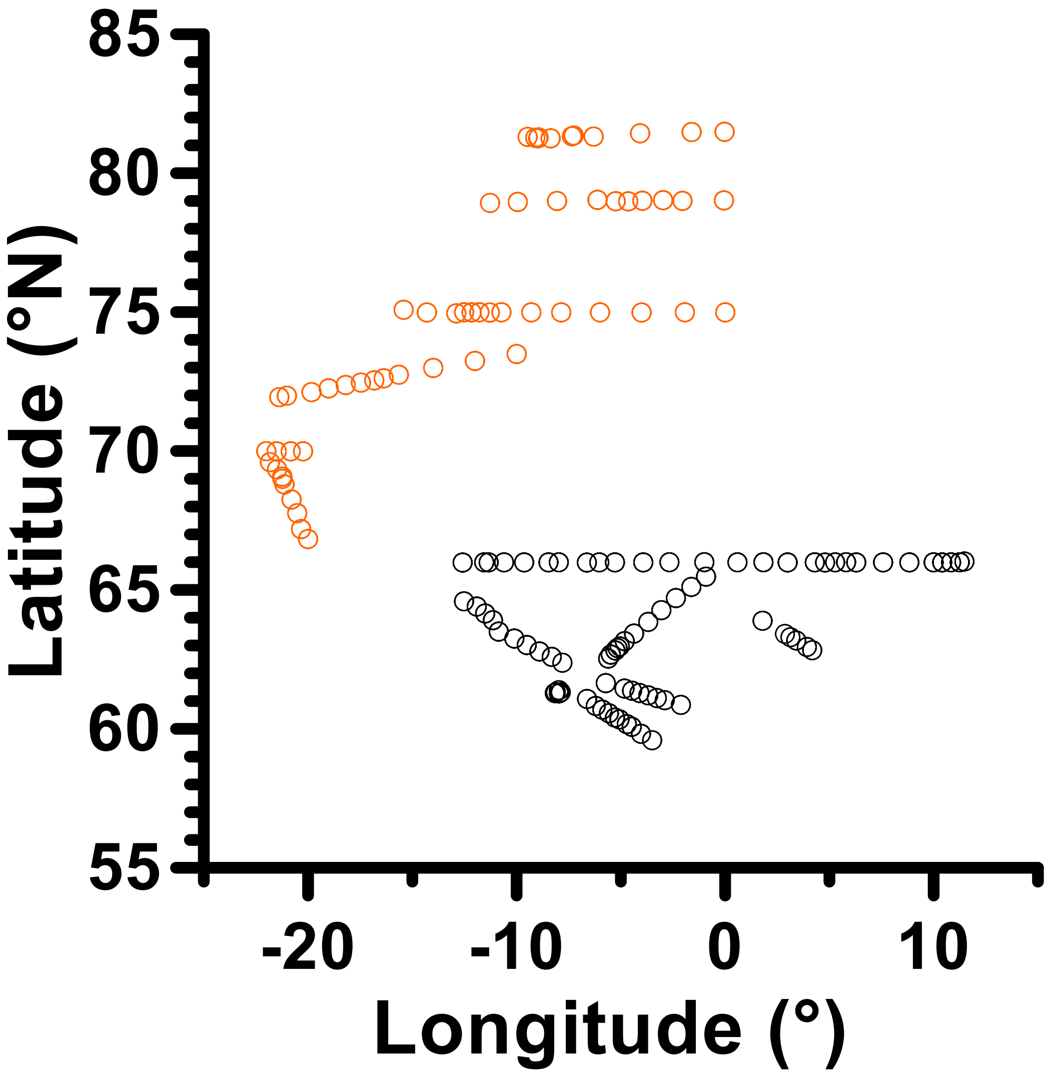

Supplementary Figure 4. The exact positions for the hydrographic stations[12] used in this study from the Norwegian Sea (black circles) and the Greenland Sea (orange circles). These hydrographic sites from the Norwegian Sea are also used to calculate the intercept 'c' in equation (1) after removing the anthropogenic carbon effect (see Methods).

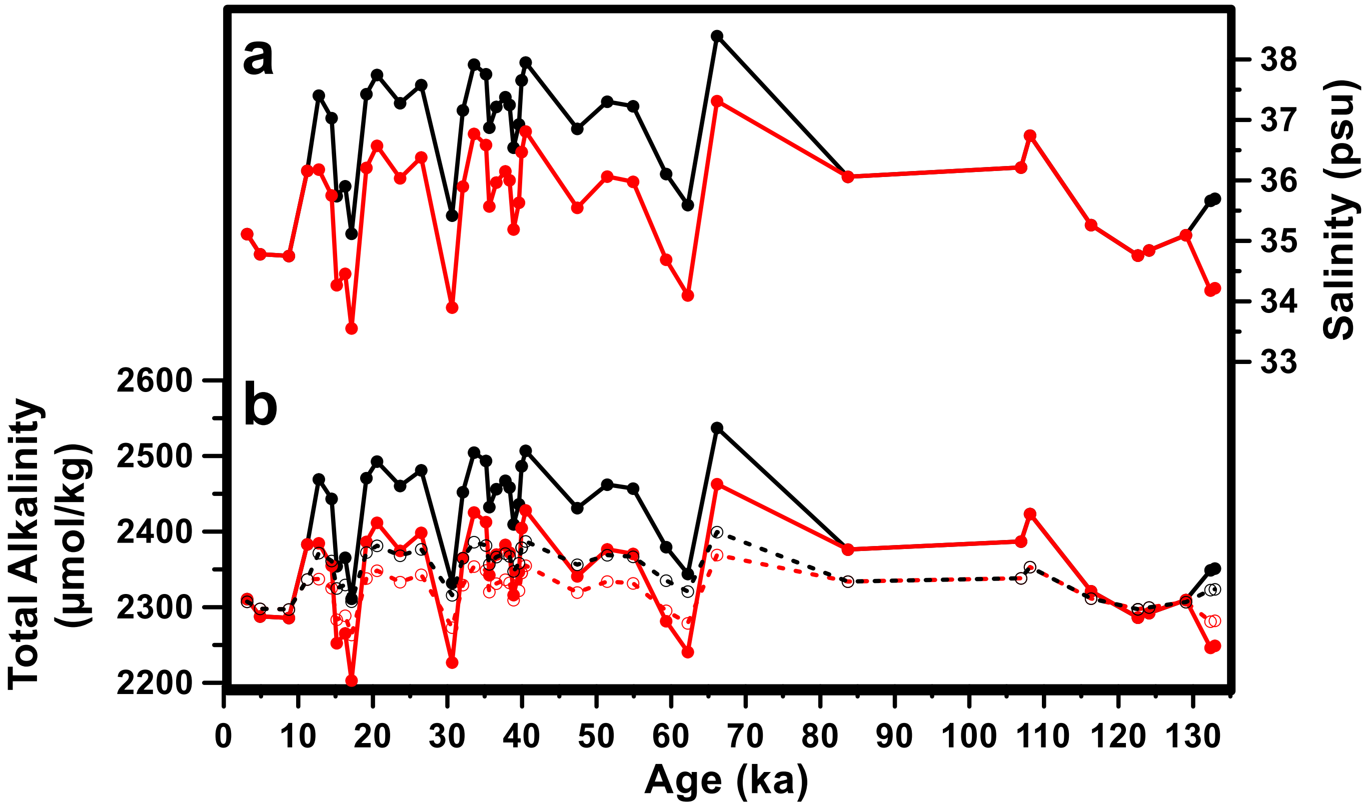

Supplementary Figure 5: Estimates of total alkalinity based on using different total alkalinity-salinity relationships. (a) Salinity reconstructions based on using modern $\delta^{18}O_{SW}$-salinity relationship for the entire record (red) or using a mixing line from Kangerdlugssuaq Fjord for the last glacial (black) (see Methods). (b) Corresponding total alkalinity reconstructions using the modern local alkalinity-salinity relationship (solid lines) or modern alkalinity-salinity relationship from the Greenland Sea (dashed lines).

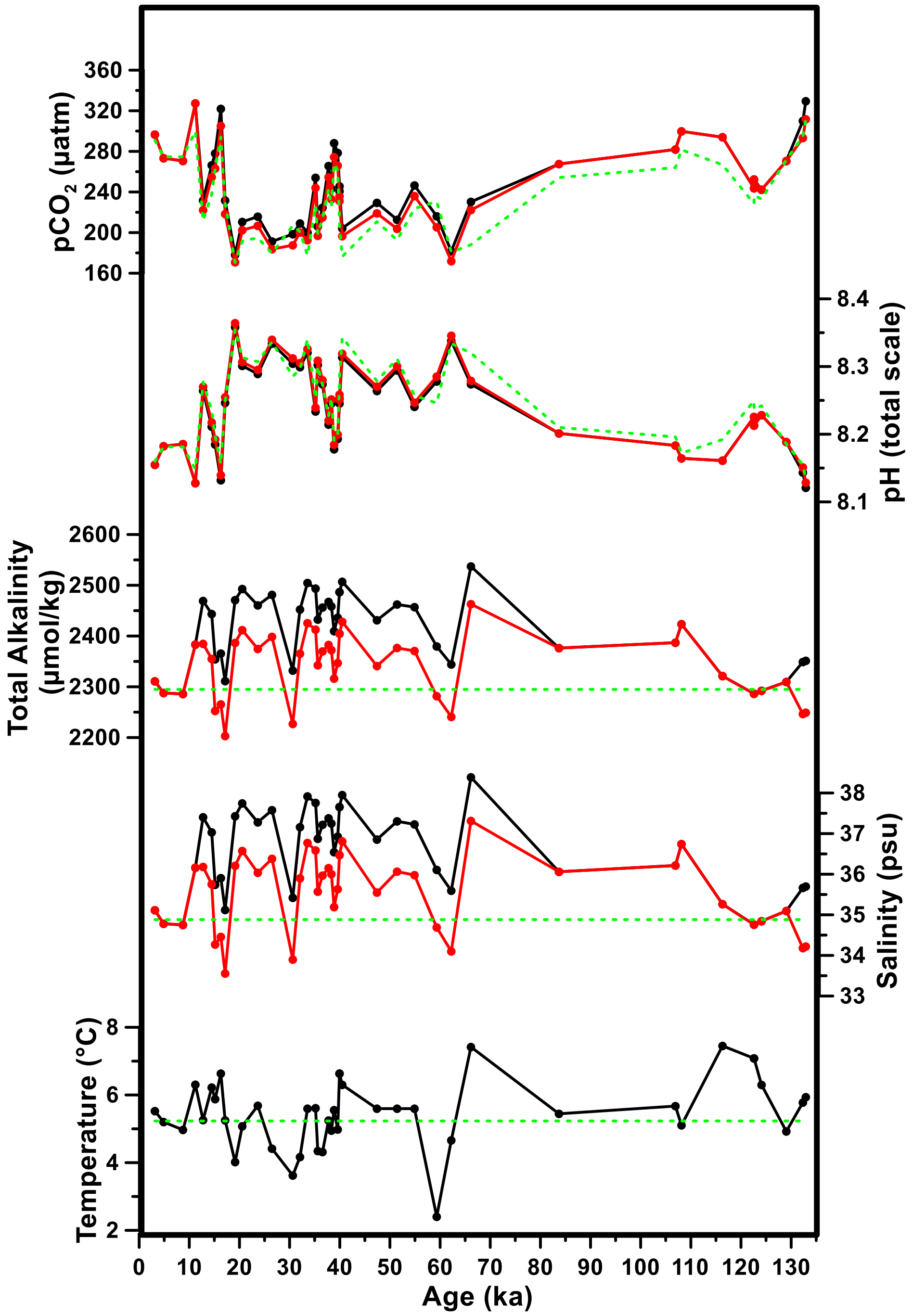

Supplementary Figure 6. Sensitivity tests of downcore pH and pCO_2 estimates to the respective uncertainties in temperature, salinity and total alkalinity. Black line and dots show temperature, salinity and total alkalinity of scenario 1, which is adopted in this study (see Methods). Green dashed lines reflect scenario 2, in which temperature, salinity and total alkalinity were kept constant. Red line and dots refer to scenario 3, in which salinity (and consequently total alkalinity) were estimated for the entire record using the modern $\delta^{18}O_{SW}$-salinity mixing model from the Norwegian Sea (see Methods). These sensitivity experiments indicate that different temperature and salinity estimates exert little effect on our pH and pCO_2 reconstructions.

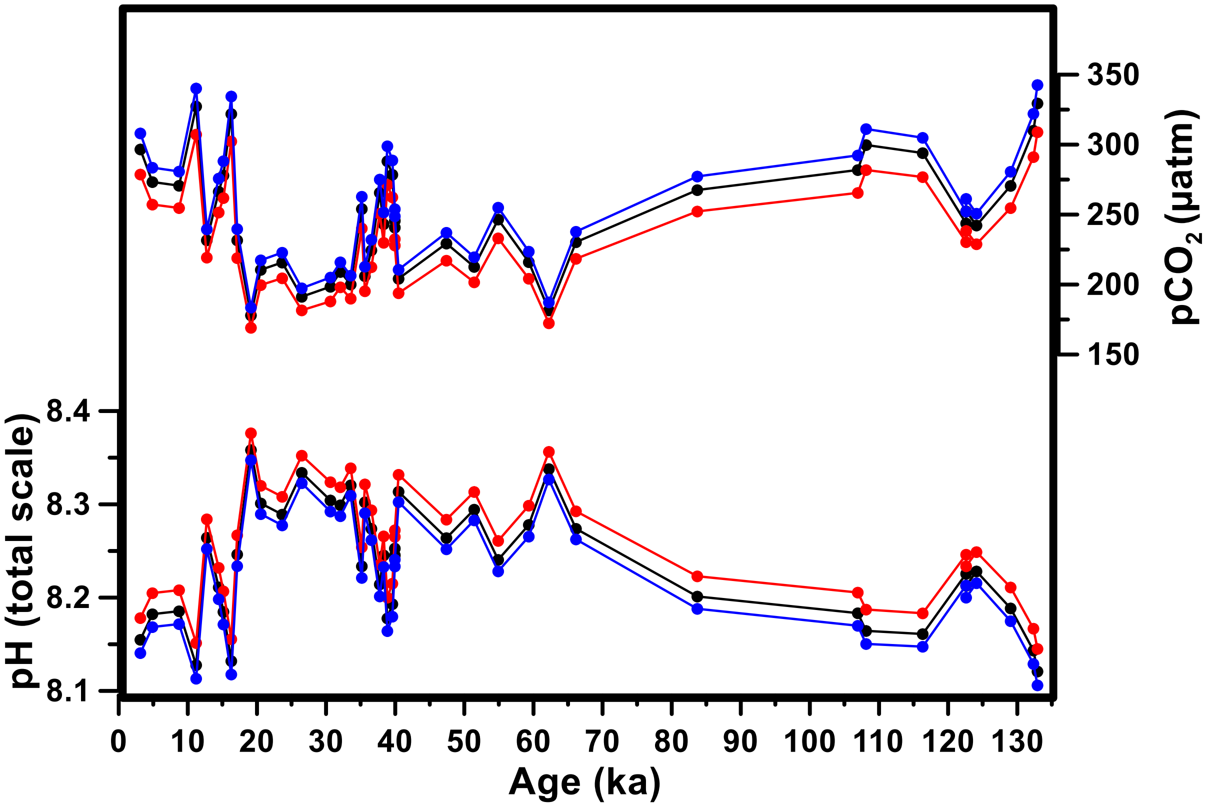

Supplementary Figure 7: A sensitivity test of the downcore pH and pCO_2 estimations for the uncertainty in the calcification depth of *N. pachyderma* including its effect on the choice of modern hydrographic data used to adjust the species-specific 'intercept' in equation (1) (see Methods). In this study, we assumed a calcification depth range from 40 to 120 m water depth (black dots and line). Alternatively, we calculated the species-specific 'intercept' based on data from 50 m water depth (red dots and line) and 200 m water depth (blue dots and line). While absolute values vary slightly, the amplitude of our downcore pCO_2 estimates varies little in response to this uncertainty.

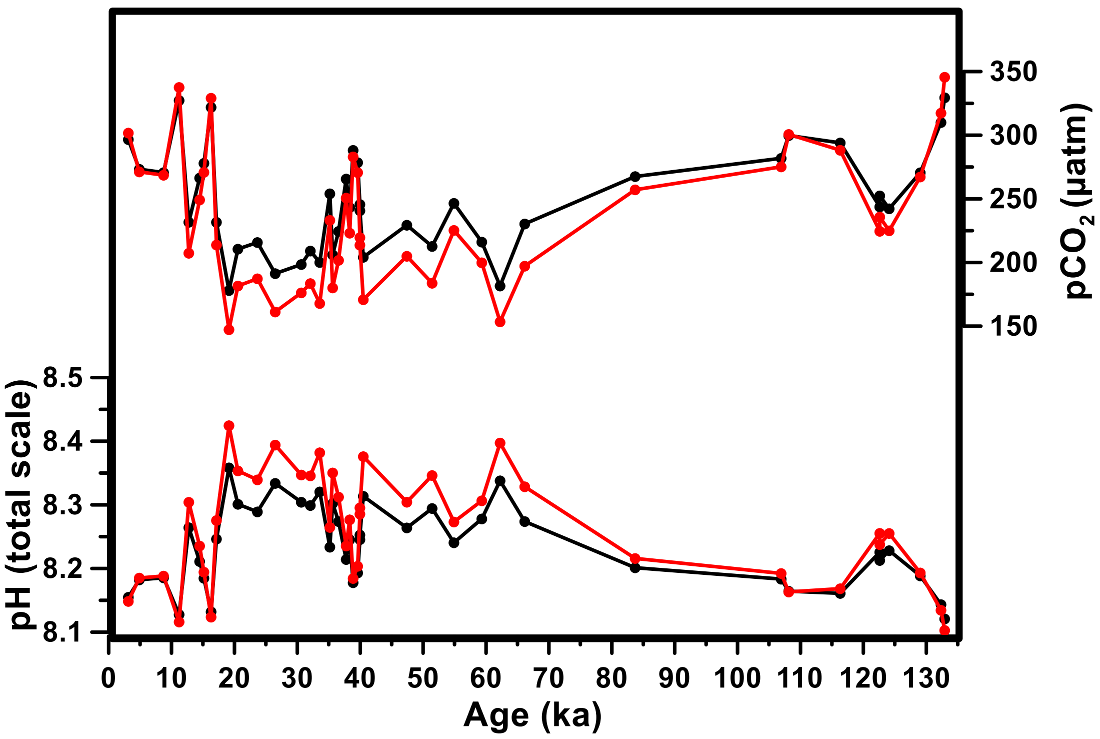

Supplementary Figure 8: Effect of different $\delta^{11}B_{CaCO_3}$ versus $\delta^{11}B_{borate}$ sensitivities on downcore pH and pCO_2 reconstructions. Black symbols and line are based on a $\delta^{11}B_{CaCO_3}$ versus $\delta^{11}B_{borate}$ slope value of 1.074. Red symbols and line reflect the same estimates using a slope value of 0.7 (see 'Sensitivity tests' section). This test shows that the relative changes discussed in this study are not affected by this uncertainty.

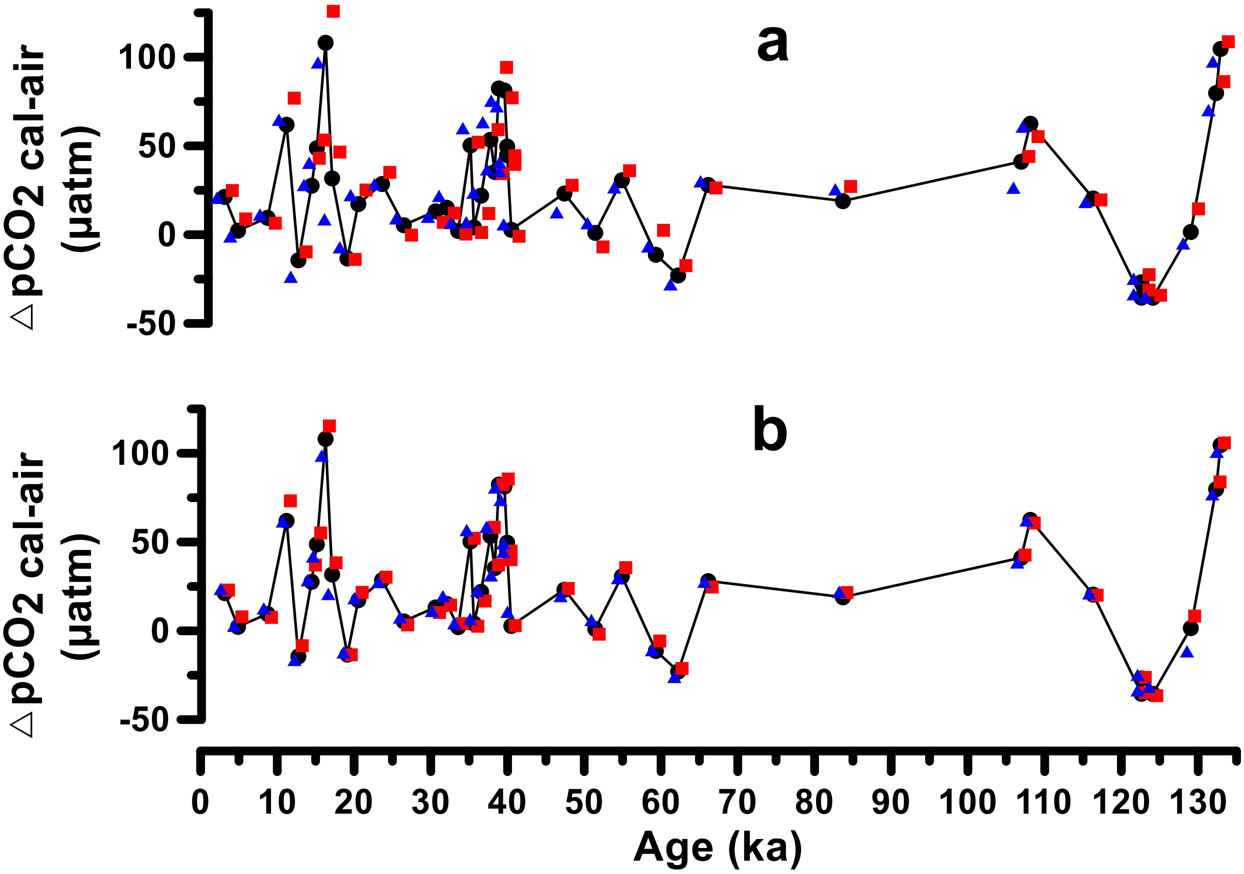

Supplementary Figure 9: Sensitivity study to the effect of uncertainty in age model on our calculated $\Delta pCO_{2cal\text{-}air}$. We added and subtracted 1000 years (a) and 500 years (b) to our age model. Red rectangles and blue triangles indicate pCO_2 scenarios when the 1000 and 500 years were added, respectively subtracted. $\Delta pCO_{2cal\text{-}air}$ based on our age model is in black line and dots for comparison.

Depth	Age	$\delta^{11}B$				$\delta^{18}O$	Mg/Ca	Seawater pCO_2	
		value	No of measure-ments	Internal uncertainty	Applied uncertainty				
(cm)	(ka)	‰		‰	‰	‰	mmol/mol	Value (µatm)	Error (µatm)
25	3.1	15.95	6	0.43	0.43	2.88	0.70	296	42
39	4.9	16.14	13	0.35	0.35	2.83	0.67	274	32
70	8.7	16.14	4	0.18	0.30	2.96	0.66	271	28
105	11.2	15.85	5	0.39	0.39	3.62	0.75	326	43
145	12.7	17.09	4	0.07	0.30	4.19	0.68	236	24
190	14.5	16.66	7	0.27	0.27	3.70	0.75	270	27
200	15.1	16.30	8	0.29	0.29	2.91	0.72	280	31
210	16.3	15.81	6	0.2	0.23	2.86	0.78	321	36
217.5	17.1	16.79	4	0.18	0.30	2.70	0.68	235	26
235	19.1	17.95	10	0.26	0.26	4.70	0.60	184	16
247.5	20.6	17.48	4	0.15	0.30	4.72	0.66	216	21
275	23.6	17.41	5	0.30	0.30	4.29	0.71	221	22
302.5	26.5	17.74	3	0.36	0.36	4.76	0.62	197	21
335	30.6	17.18	6	0.16	0.23	3.43	0.57	203	19
345	32.1	17.30	4	0.09	0.30	4.39	0.61	214	21
360	33.6	17.79	4	0.35	0.35	4.54	0.70	206	21
385	35.2	16.84	4	0.17	0.30	4.41	0.70	258	26
392.5	35.6	17.34	9	0.36	0.36	4.10	0.62	211	24
410	36.6	17.05	6	0.32	0.32	4.29	0.62	229	24
432.5	37.8	16.58	6	0.33	0.33	4.25	0.68	269	32
442.5	38.3	16.85	5	0.03	0.26	4.27	0.66	247	24
450	38.9	16.23	7	0.22	0.22	3.60	0.70	290	28
457.5	39.6	16.32	4	0.18	0.30	4.01	0.66	280	31
465	39.9	17.10	3	0.34	0.34	4.08	0.78	251	27
465	39.9	17.18	3	0.26	0.34	4.08	0.78	246	26
472.5	40.5	17.82	5	0.27	0.27	4.36	0.75	211	18
550	47.4	17.11	3	0.13	0.34	3.81	0.70	234	26
577	51.4	17.46	4	0.15	0.30	4.02	0.70	218	21
605	54.9	16.88	4	0.13	0.30	3.96	0.70	251	26
665	59.3	16.77	4	0.26	0.30	4.00	0.51	219	24
680	62.2	17.72	4	0.29	0.30	3.27	0.64	187	19
695	66.2	17.56	4	0.43	0.43	4.48	0.84	237	29
780	83.7	16.42	4	0.37	0.37	3.77	0.69	270	32
881	106.9	16.28	4	0.28	0.30	3.75	0.71	284	29
889	108.1	16.06	5	0.29	0.29	4.25	0.67	300	31
931	116.3	16.24	10	0.27	0.27	2.74	0.84	295	28
966	122.6	16.80	4	0.24	0.30	2.32	0.81	247	23
966	122.6	16.67	3	0.48	0.48	2.32	0.81	256	36
981	124.1	16.73	4	0.22	0.30	2.53	0.75	246	23
1027	129.0	16.18	3	0.34	0.34	2.98	0.65	272	31
1067	132.3	15.90	5	0.26	0.26	2.49	0.71	309	35
1075	132.9	15.73	4	0.30	0.30	2.68	0.72	329	40

Supplementary Table 1: Data used for pCO_2 reconstruction. The grey-shaded samples are those repeated for boron isotope analyses after using the reductive-oxidative cleaning method[13]. For the entire $\delta^{11}B$ record, we used the oxidative cleaning method[14]. The two cleaning methods yield indistinguishable results. Temperatures highlighted by an asterisk (*) indicate that there are no Mg/Ca measurements at these exact depths and a temperature of 6 °C has been assumed (equivalent to Mg/Ca of ~0.70 mmol/mol according to the calibration applied herein). The internal uncertainty for $\delta^{11}B$ analyses is compared to the long-term reproducibility of an in-house vaterite standard (±0.34‰ for n=3 to ±0.19‰ for n=10) and the larger of the two uncertainties is reported.

Depth cm	B/Ca µmol/mol	Mg/Ca mmol/mol	Al/Ca µmol/mol	Mn/Ca µmol/mol	Fe/Ca µmol/mol	Cd/Ca µmol/mol	DTPA step
215	77.0	0.695	4	92	14	0.0071	Yes
215	76.4	0.922	118	121	41	0.0074	No
345	87.2	0.607	2	89	35	0.0024	Yes
345	87.6	0.646	168	97	30	0.0026	No
490	90.3	0.788	43	25	48	0.0008	Yes
490	82.7	1.272	297	30	227	0.0013	No
670	78.6	0.671	9	75	36	0.0029	Yes
670	78.2	0.725	64	84	46	0.0030	No
682.5	86.6	0.583	2	31	21	0.0024	Yes
682.5	84.1	0.672	28	35	26	0.0026	No

Supplementary Table 2: A test for the influence of the alkaline chelation (using DTPA) step on the Cd/Ca, B/Ca and Mg/Ca.

Supplementary References

1. Hoff, U., Rasmussen, T. L., Stein, R., Ezat, M. M., & Fahl, K. Sea ice and millennial-scale climate variability in the Nordic seas 90 ka to present. *Nat. Commun., 12247:* doi: 10.1038/ncomms12247 (2016).
2. Ezat, M. M., Rasmussen, T. L., Groeneveld, J. Reconstruction of hydrographic changes in the southern Norwegian Sea during the past 135 kyr and the impact of different foraminiferal Mg/Ca cleaning protocols. Geochem. Geophys. Geosyst., 17, doi:10.1002/2016GC006325 (2016).
3. Ezat, M. M., Rasmussen, T. L., & Groeneveld, J. Persistent intermediate water warming during cold stadials in the southeastern Nordic seas during the past 65 k.y. *Geology* **42,** 663–666 (2014).
4. Rasmussen, T. L., Thomsen, E., Labeyrie, L., & van Weering, T. C. E. Circulation changes in the Faeroe-Shetland Channel correlating with cold events during the last glacial period (58–10 ka). *Geology* **24,** 937–940 (1996).
5. Rasmussen, T. L., Thomsen, E., Kuijpers, A., & Wastegård, S. Late warming and early cooling of the sea surface in the Nordic seas during MIS 5e (Eemian Interglacial). *Quat. Sci. Rev.* **22,** 809–821 (2003).
6. Rasmussen, S. O., *et al.* A stratigraphic framework for abrupt climatic changes during the Last Glacial period based on three synchronized Greenland ice-core records: refining and extending the INTIMATE event stratigraphy. *Quat. Sci. Rev.* **106,** 14–28 (2014).
7. Svensson, A., *et al.* A 60 000 year Greenland stratigraphic ice core chronology. *Clim. Past* **4,** 47–57 (2008).
8. Thornalley, D. J. R., Elderfield, H., & McCave, I. N. Reconstructing North Atlantic deglacial surface hydrography and its link to the Atlantic overturning circulation. *Global Planet. Change* **79,** 163-175 (2011).
9. Yu, J., Thornalley, D. J. R., Rae, J. W. B., & McCave, N. I. Calibration and application of B/Ca, Cd/Ca, and δ^{11}B in *Neogloboquadrina pachyderma* (sinistral) to constrain CO_2 uptake in the subpolar North Atlantic during the last deglaciation. *Paleoceanography* **28,** 237-252 (2013).
10. Foster, et al., Interlaboratory comparison of boron isotope analyses of boric acid, seawater and marine $CaCO_3$ by MC-ICPMS and NTIMS. *Chemical Geology* **358,** 1-14 (2013).
11. Farmer, J. R., Hönisch, B., & Uchikawa, J. Single laboratory comparison of MC-ICP-MS and N-TIMS boron isotope analyses in marine carbonates. *Chemical Geology*, doi: http://dx.doi.org/10.1016/j.chemgeo.2016.11.008 (2016).
12. Key, R. M., et al. The CARINA data synthesis project: introduction and overview. *Earth Syst. Sci. Data* **2,** 105-121 (2010).
13. Pena, L. D., Calvo, E., Cacho, I., Eggins, S., & Pelejero, C. Identification and removal of Mn-Mg-rich contaminant phases on foraminiferal tests: Implications for Mg/Ca past temperature reconstructions. *Geochem. Geophys. Geosyst.* **6,** doi: 10.1029/2005GC000930 (2005).
14. Barker, S., Greaves, M., & Elderfield, H. A study of cleaning procedures used for foraminiferal Mg/Ca paleothermometry. *Geochem. Geophys. Geosyst.* **4,** doi: 10.1029/2003GC000559 (2003).
